# A morphological, morphometric and geochemical characterization of the El Jobo projectile points – diversity and significance in early human populations across the Americas

**DOI:** 10.1007/s12520-025-02296-2

**Published:** 2025-08-16

**Authors:** Diego Vargas, Kévin Le Verger, Guido L. B. Wiesenberg, Carlos von Büren, Jorge D. Carrillo-Briceño, Arturo Jaimes, Marcelo R. Sánchez-Villagra

**Affiliations:** 1 Department of Anthropology of the Americas, University of Bonn, Oxfordstraße 15, 53111 Bonn, Germany; 2 Centro de Antropología, Instituto Venezolano de Investigaciones Científicas (IVIC), Carretera Panamericana, Km 11, San Antonio de los Altos, 1204 Venezuela; 3 Department of Paleontology, University of Zurich, Karl-Schmid-Strasse 4, 8006 Zurich, Switzerland; 4 Department of Geography, University of Zurich, Winterthurerstrasse 190, 8057 Zurich, Switzerland

**Keywords:** Clovis, Paleoindian, Weaponry, Venezuela, Caribbean, Monte Verde

## Abstract

The El Jobo projectile points represent a distinctive design innovation of Late Pleistocene Neotropical human groups. This technology, characterized by its fusiform/lanceolate shape, has been recorded mainly in northwestern Venezuela in a variety of geographical areas and in association with megafauna killing/butchering sites. To characterize it, address its significance, and analyse possible continental relationships, four consecutive approaches were conducted. A representative sample of El Jobo projectile points was subject to technological approximation, morphological classification, outline-based geometric morphometric analysis, and elemental composition analysis. Six morphological types were recognized, for which a common series of manufacture steps was hypothesized. Mainly collateral and irregular flaking patterns were identified, also recording new basal variability, including fluting, fluting-like and reconfiguration techniques. The four most representative morphological types were subjected to morphometric analysis. Elliptic Fourier and Principal Component analyses identified at least three clusters, with width variation mainly distinguishing their shapes, and statistical tests determined significative differences between the main morphological types. The elemental analysis revealed the main use of quarzitic rocks, with variations in elemental composition indicative of diverse sources. The observed variability and cumulative evidence of El Jobo projectile points suggests their adaptation to diverse hunting strategies. The wide geographic distribution of tools and the morphometric comparison with a Monte Verde projectile suggest potential long-distance connections of El Jobo with other projectile point technologies across the continent.

**Supplementary Information:**

The online version contains supplementary material available at 10.1007/s12520-025-02296-2.

## Introduction

Northwestern Venezuela constitutes an outstanding region for the archaeological study of late Pleistocene and early Holocene human cultural evolution given its geographic location, arid climate, and lower rates of sediment deposition (Sánchez-Villagra et al. 2024). Sites documenting exploitation of megafauna have been recorded in this region (Bampi et al. 2022; Carlini et al. 2022; Jaimes et al. 2024b). Regarding the material culture, while artifacts of bone and ivory have been found, most of the evidence recovered is composed of stone tools (Carrillo et al. 2025; Ochsenius and Gruhn 1979; Rouse and Cruxent 1956; Szabadics 1997).

Different lithic technologies have been reported for northern Venezuela, such as Clovis (Jaimes et al. 2024a; Pearson and Ream 2005) and Fishtail (Garzón 2019; Nami 2016); as well as local technologies known as Camare, Las Lagunas, El Jobo and Las Casitas, the latter four representing a long debated technological sequence (Jaimes et al. 2024a; Oliver and Alexander 2003). El Jobo technology stands out, due to the large amount of collected materials (Rouse and Cruxent 1956) and its association with megafauna hunting/butchering across key sites. Such sites include Taima-Taima (Ochsenius and Gruhn 1979), Cucuruchú (Cruxent 1970), Muaco (Royo y Gómez 1960), and El Vano (Jaimes et al. 2024b). El Jobo is a technology of historical significance given its early dated record of ‘pre-Clovis’ presence in the Americas in the 1970 s (Bryan et al. 1978; Lynch 1990; Politis et al. 2009). The general pre-Clovis chronology—and stratigraphy—proposed by Bryan and Gruhn (1979) for Taima Taima has been verified by the acquisition of new dates from dental enamel of Taima-Taima mammals (Carlini et al. 2022; Carrillo-Briceño et al. in prep; Supplementary material 1).

While initially, El Jobo projectile points were characterized as a homogeneous technology (Jaimes 2005; Rouse and Cruxent 1956), there is considerable morphological variation (Admiraal 2013; Carrillo 2015: figs. 136–37), including the fluting technique in the technological repertoire of the El Jobo assemblage (Jaimes et al. 2024a). The overall shape of El Jobo projectile points is bi-pointed fusiform, with biconvex or diamond-shaped sections (Cruxent and Rouse 1958; Jaimes 1999; Jaimes et al. 2024a). With ‘fusiform’, we mean an elongated, ellipsoid form, with the two ends narrower than the center (Admiral 2013, p 13). This fusiform shape, as described below, is rather lanceolate, or leaf-shaped in some variations of El Jobo. El Jobo points can have great thickness, sometimes reaching the same dimension as the width (Admiraal 2013). They can exhibit also a high diversity of bases, including pointed, rounded, reduced, fractured and reworked bases, as well as thinning in the medial area to generate a simulated stem (= socketed shaft of Admiral 2013, p. 15) or a clear restricted stem (Cruxent and Rouse 1958; Jaimes 1999).

**Fig. 1:**
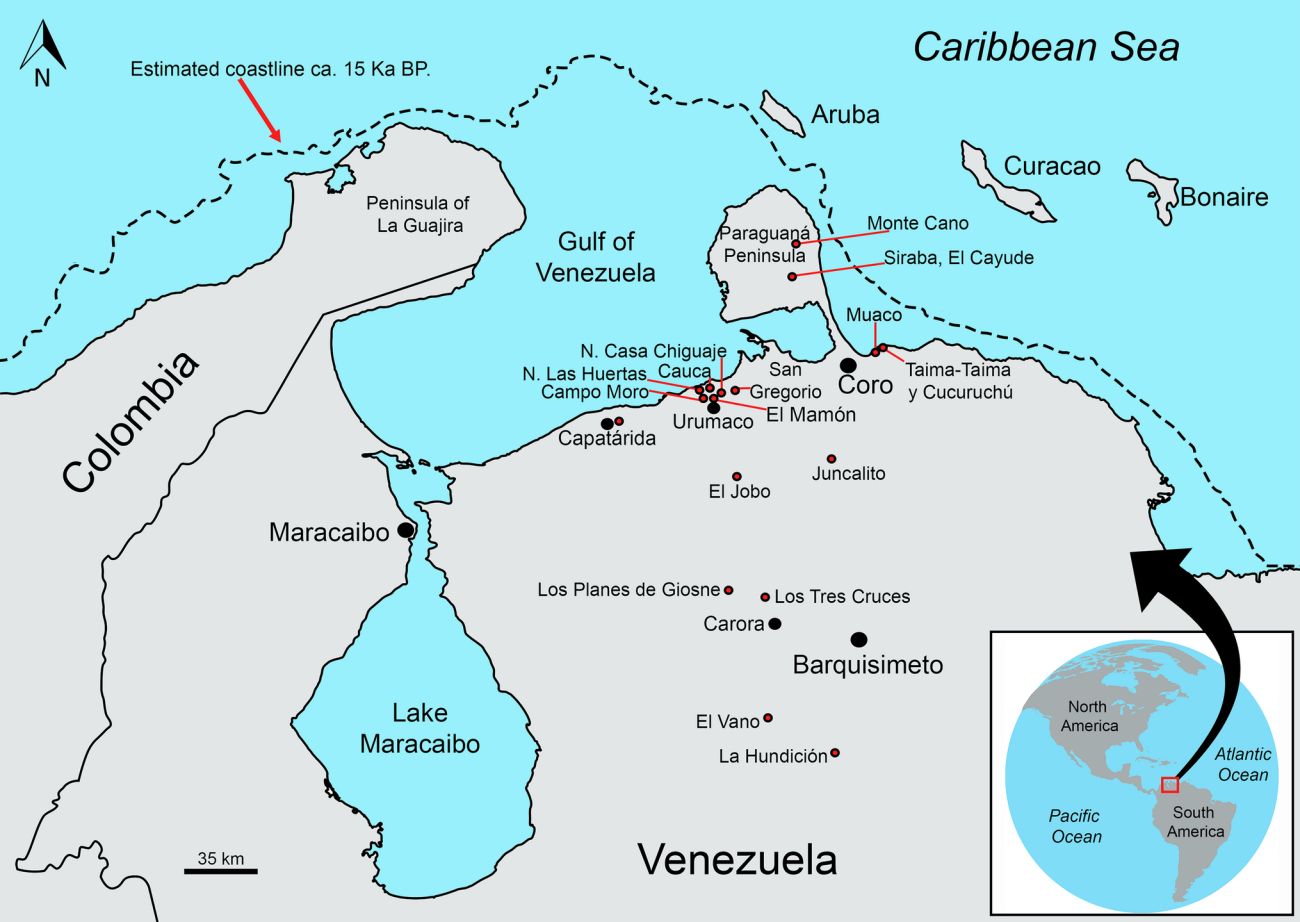
Geographical location of archaeological sites with El Jobo projectile points in northern Venezuela. Map and estimated coastline based on GEBCO (2024) Grid

The first studies of El Jobo in Falcón State (Fig. 1) reported that the raw material was quartz sandstone (Cruxent and Rouse 1958). Later studies reported lithic workshops in the locality of Monte Cano, in the Paraguaná Peninsula, where the stone used was milky quartz (Rodríguez and Morganti 1984). Similarly, solitary projectile points have been collected, which were made from other types of rock such as black, red, and yellow chert, basalt, or rock crystal, among others (Jaimes 1999). Sedimentary rocks such as sandstone appear in the region in various forms: as outcrops (boulders), in veins, and as river cobbles. We performed a geochemical evaluation of the samples analyzed here as a quantitative approximation to the variation in the rock-source of El Jobo points. We hypothesize that the element analysis will reveal different compositions, paralleling the morphological variation we quantify in this work.

El Jobo technology has been identified in other regions outside Venezuela (Cooke 2005; Waters et al. 2018), suggesting potential long-distance transmission of technological traits throughout the continent at the end of the Pleistocene. There is a continuity of gradient of form between El Jobo and Clovis technology (Jaimes et al. 2024a; see below), and relations with the thickened body lanceolate points from North America (Admiraal 2013; Rouse and Cruxent 1956) lends support to this proposal, and make quantification of the features and variations of the El Jobo points timely. Our study serves to examine the implications of El Jobo variation, and the possible relations of lithic technologies across the Americas. This includes investigating potential but never quantified similarities with the projectiles found at Monte Verde II in Chile (Dillehay 2000, 2019: fig. 7; Waters et al. 2018). The diverse subjects brought in our study provide to El Jobo technology an integrated approach to study Late Pleistocene lithic technology (Huidobro Marín et al. 2023; González-Varas et al. 2025).

### Environmental and chronological context

Known sites associated with El Jobo technology were situated at greater distances from the present coast due to lower sea levels during the Pleistocene, making them completely “inland sites” (Fig. 1). While the southernmost sites have been discovered in lower mountains (less than 700 m above sea level), the majority of sites are situated in valleys, depressions, and coastal plains between 10 and 500 m above sea level.

The Lara-Falcón Formation region of Venezuela is mainly characterized by xerophytic shrubland vegetation, typically of most of the northern Falcon state (Ochsenius 1980). While early Spanish colonization with the introduction of goats may have had some environmental impact, the dominance of xerophytic shrubland is primarily a result of the region's arid climate and is not solely attributable to human activity (Jaramillo et al. 2020). Historical records confirm that this vegetation type was prevalent even before European arrival.

The presence of megafauna at El Jobo sites and dietary inferences from the Pleistocene megaherbivores suggest a more humid past and a diverse paleoenvironment at the time. Proboscideans likely consumed grasses, while browsing taxa such as *Palaeolama*, *Xenorhinotherium*, *Mixotoxodon*, and *Eremotherium* indicate the presence of shrubs and trees. A quantitative assessment of the megafauna's dietary ecology suggests that the most probable environment was an open savannah with scattered trees and shrubs (Carrillo et al. in prep; Wilson et al. 2024).

According to Ochsenius (1979, 1980), the Falcon region likely experienced a negative water balance. This arid environment probably made the Taima-Taima, Muaco, Cucuruchú, and El Vano waterholes, which were likely permanent, crucial oases for animals during dry seasons, consequently serving as hunting grounds (Aguilera 2006; Carrillo-Briceño 2015; Cruxent 1970, 1979; Jaimes 2003; Royo and Gómez 1960).

**Fig. 2:**
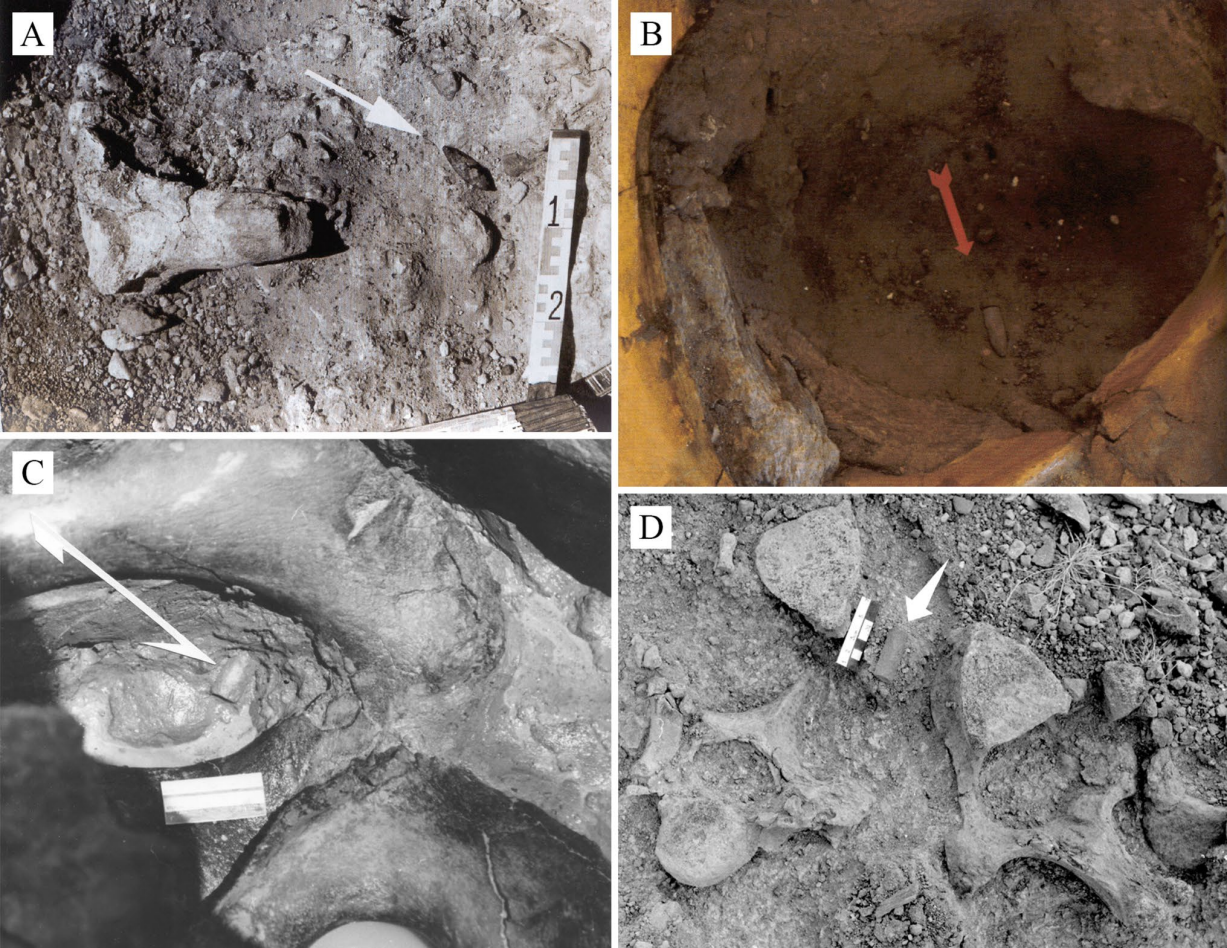
In situ El Jobo projectile points. **A**) Association of Cucuruchú excavation, modified after Aguilera (2006). **B**) Association of Taima-Taima excavation of 1974, modified after Aguilera (2006). **C**) Taima-Taima excavation of 1976, archive of the Archaeology Laboratory of the IVIC. **D**) El Vano excavation, photograph by AJ

Although El Jobo projectile points have been found in these sites (Fig. 2), only three points have been accurately dated so far, specifically the ones of the Taima-Taima and El Vano sites. At Taima-Taima, two projectiles were recovered from the pelvic regions of two distinct gomphotherids (Ochsenius and Gruhn 1979; Fig. 2B-C). The dates obtained for the stratigraphic unit containing this evidence range from 13,487 to 17,809 calyBP (Supplementary material 1). In the case of El Vano, dated from 12,616 to 12,760 calyBP, the only known killing site of an *Eremotherium* on the continent, a point was located between the dorsal vertebrae of the animal (Jaimes et al. 2024a, b; Fig. 2-D).

## Materials

For sampling selection, a partial revision (approximately one-third) of the archaeological collection from the current geographic designation for the"El Jobo"region, *i.e.*, Pedregal River basin, was conducted. This collection was initially compiled by J. M. Cruxent between the 1950 s and 1970 s and is presently curated at the Archaeological Collection of the Instituto Venezolano de Investigaciones Científicas (IVIC). A total of 1,342 projectile point fragments associated with the El Jobo tradition were identified. Of these, 106 points were selected based on their level of completeness for the analysis. To further document lithic technologies from this neotropical region, four projectile points from the Los Planes de Giosne site (Jaimes 1999), five from the Juncalito site (Jaimes et al. 2024a), and a single point from El Vano (Jaimes et al. 2024b: fig. 7) were also incorporated into this study, all of which are curated in the same archaeological collection. Additionally, photos and 3D models of preforms from the same collection were used to complement the descriptions, as well as a 3D model of a Monte Verde point provided by Antonio Pérez-Balarezo.

The different points, and typologies, occur in the same sites, but there is a lack of stratigraphical control in most cases as to discern their temporal co-occurrence.

## Methods

Four complementary approaches were conducted for the selected sample to characterize El Jobo projectile point technology: 1) A reconstruction of the process of manufacture; 2) An initial classification according to morphological attributes of the samples; 3) An outline based geometric morphometric analysis to extract and compare point shape of each previously classified group; 4) An elemental analysis to provide an approximation of the diversity in rock composition. Finally, an additional morphometric analysis investigates the shape of a projectile point from Monte Verde and El Jobo points.

### Technological approximation

The type of shaping flaking pattern and edge retouch were registered through macroscopic observation, following Admiraal (2013: Fig. 1) categories for the lanceolate projectile points (e.g. El Jobo), and de Sousa and Okumura (2020) categories for the stemmed triangular points (e.g. Las Casitas, see below). The description of the manufacturing process of the projectile points was based both on the analysis of the initial sample and on the examination of preforms of different manufacturing stages.

### Morphological types

The whole outline and medial sections of the samples were identified, and the length, width, and thickness of all projectile points were measured using a 150 mm Mitutoyo digital caliper. The fineness index (Width:max. thickness; Mesfin et al. 2020) was calculated and plotted according to length. Preliminary typologies were established based on the general shape of the samples and on the descriptions provided in previous studies (see Jaimes et al. 2024a).

### Whole-outline geometric morphometrics

Prior to the analysis, a set of 33 samples was digitized using a Nikon SWM ED IF Aspherical Micro 1:1, enabling the production of an image of their frontal face with a resolution of 3000/4496 pixels and 300 dpi. This specific set of samples was selected based on their completeness. After binarization to facilitate automatic extraction of outlines using Adobe Photoshop, all samples were then assembled into a projectile point plate provided with a scale to produce a single Thin-Plate Spline file using tpsUtil. With this new file, we performed the protocol provided by Matzig et al. (2021), using the *Momocs* (Bonhomme et al. 2014) and the *'outlineR'* (Matzig 2021) packages in the R software environment (R Core Team 2020), to extract the shape coordinates for each single artefact and to combine all of them. Based on this recombined dataset, we performed an elliptic Fourier analysis for which we previously determined the optimal number of harmonics (= 24) to capture a maximum of the shape of the projectile points (Caple et al. 2017). As a prerequisite to the analysis, we also performed a normalization of the coefficients to align the different harmonics and thus remove the effect of size and rotation (Claude 2008).

To analyze the differences in shapes of the groups determined during the preliminary classification, we followed the protocol of Matzig et al. (2021). First, we performed a Principal Components Analysis (PCA). By investigating the morphospace of the projectile points, we can identify on the first axes whether the various groupings resulting from our identifications are found in the form of isolated clusters. In addition, we extracted shape differences along each of the main axes to identify which shape variations support the distinction between potential clusters. Then, to compare the relationship between each sample, we computed the distance matrix derived from the PC scores over the entire shape variance from which we applied hierarchical clustering (HC) with the Ward.D2 method (Murtagh and Legendre 2014). This dissimilarity analysis enables us to determine the relationship between each of the samples within the morphospace and thus measure the robustness of the PCA clusters. Based on the number of clusters identified in the dendrogram, we extracted the mean shape for each to compare the major differences in terms of shape and identify the correspondence between the samples in each group and their distribution in the clusters of the dendrogram. As a final exploration of shape variation, we also computed the shape disparity (i.e., the amount of total morphological variation, sensu Guillerme 2018) for each of our groups from the PC scores, bootstrapping the PCA data 1000 times. To assess whether the groups are statistically different according to their shape disparity, we performed a pairwise Wilcoxon signed-rank test with Bonferroni correction. Disparity analysis and statistical tests were conducted using the *dispRity* package (Guillerme 2018). Araujo et al. (2023) discussed this approach as a ‘benchmarking’ one in the study of lithic tools.

In an additional analysis, we examined the shape similarities between the studied projectile points from Venezuela and a sample from Monte Verde. We reran the morphometric analysis on a reduced set (excluding three Jobo arrow points, see result), this time including a Monte Verde (Dillehay et al. 2019) and a Los Planes projectile point (Museo Arqueológico de Quíbor MAQ-A-1236; Jaimes 1992). For further comparison, a selection for each Jobo cluster was based on a compromise between a position close to the consensus shape of each cluster and a sufficient distance from the other selected arrow points to optimally mark differences in shape. For the detailed shape comparison between the Monte Verde arrow point and the selected Jobo arrow points, we quantified differences using 3D landmarks of type 3. After a generalized Procrustes alignment, the shapes of each Jobo arrow point were superimposed separately with the Monte Verde arrow point, and we visualized vectors showing the main variations in each of these comparisons on one face and one edge.

### Elemental composition analysis

A total of 50 projectile points were selected for elemental analysis, including the majority artefacts used in the geometric morphometric analysis. The elemental composition was performed on intact objects via surface X-ray fluorescence analyses using a Thermo Fisher Scientific Niton XL5 device. The measurements were taken using the “mining” calibration provided by the manufacturer. Daily self-tests and measurements of laboratory internal reference samples ensure proper instrument function. As the surfaces of the objects were often not flat, multiple analyses (at least five) were performed per object. Out of these analytical replicate analyses, three measurements were selected that yielded the most uniform results and outliers were omitted. Outliers were determined based on exceptional high or low values of single element concentrations of multiple elements and/or the balance, the latter being the amount of non-measurable elements. Mean values and standard errors of the mean were calculated.

We also applied non-destructive magnetic susceptibility measurements on the samples using KT-10 v2 Magnetic Susceptibility Meter with a circular coil. However, as the circular coil of the instrument and the uneven surface of the projectile points received highly variable readouts, we decided to state only if the projectile points showed a positive readout or no magnetic properties; the order of magnitude of magnetic susceptibility was thus omitted in our study.

## Results

### Manufacturing of El Jobo projectile points

**Fig. 3:**
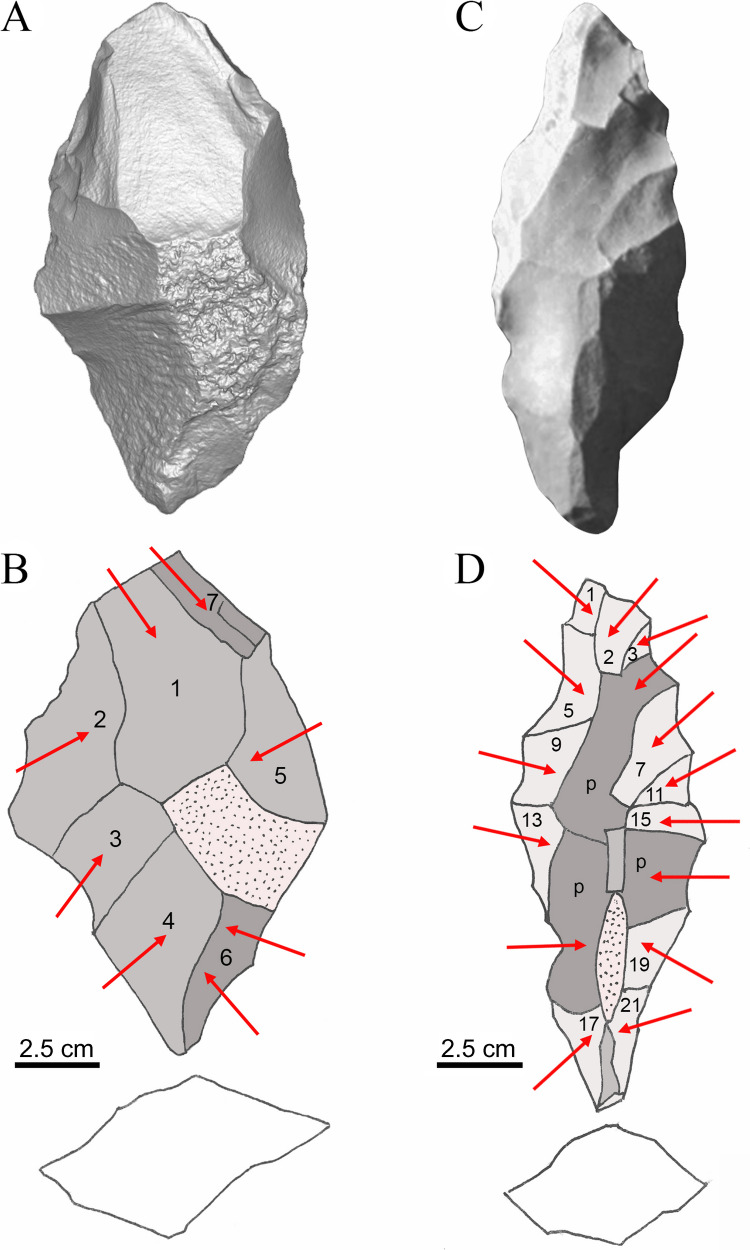
**A** 3D model of a preform in its initial stage. **B** The dotted facet shows remnants of the piece's original cortex; the order of the numbers indicates the initial roughing sequence of the preform; the light gray color represents the first stage of preforming, and the darker gray represents the start of the next stage. **C** Photograph of a bifacial preform in its third stage of forming. **D** Reconstruction of the forming processes, where P (dark gray) shows the negatives from the previous stage; the numbers show the reduction sequence applying the"continuous alternating"technique on both faces, obverse and reverse, over the light gray color. The arrows indicate the direction of the blows. Note the decrease in scarring on preform in D. Reference scale: 2.5 cm

The initial reduction of the matrix is done with direct percussion (Nami 1994), generating negatives up to 3 cm wide and 5 cm long in the reduction of the sides of the piece, seeking to reduce the central ribbing of the piece. This phase can have between 12 and 15 detachments per face in a complete round. There can be up to 5 initial rounds (Fig. 3A-B). Afterwards, the intensity of the formatting blows is reduced, as well as those aimed at reducing the zone of maximum thickness on the central axis of the piece, and whose total dimensions are already about 20% less than the original piece.

**Fig. 4:**
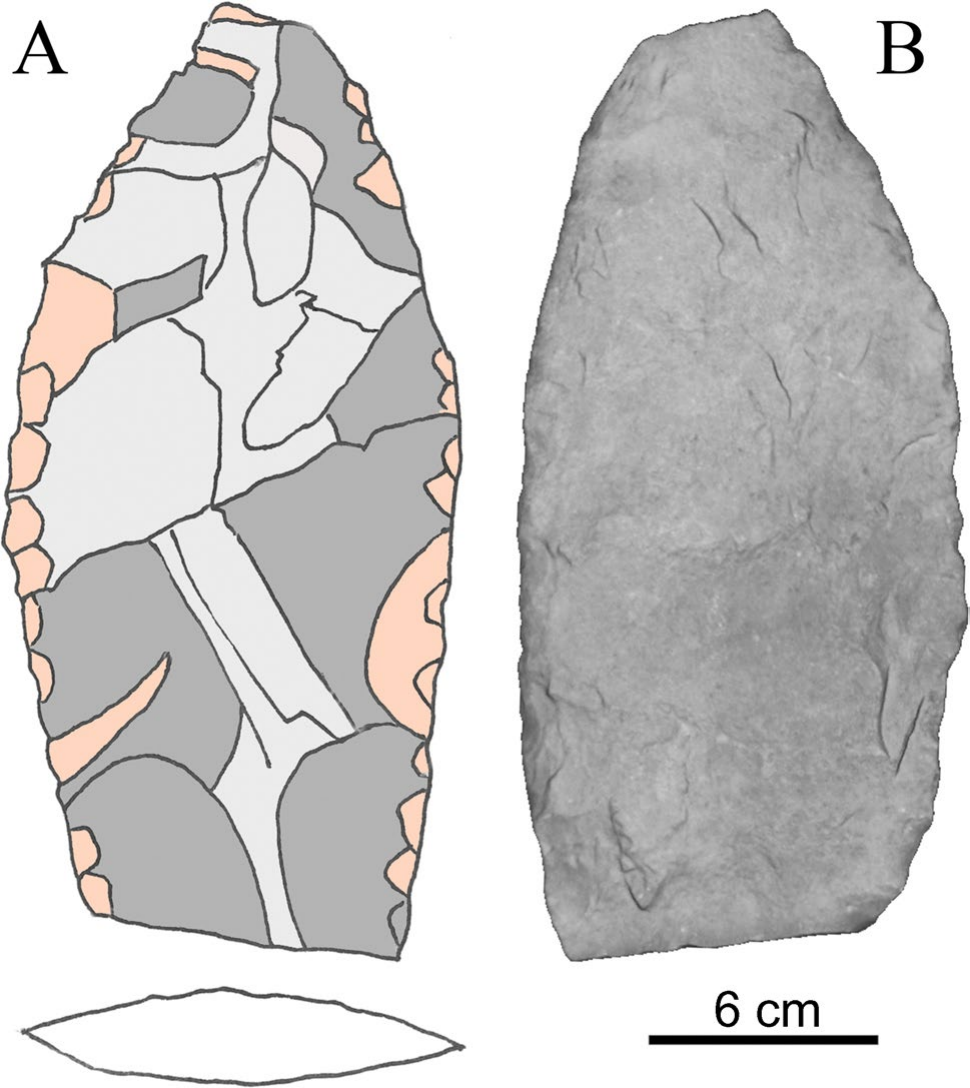
**A** Bifacial preform in the fourth stage of reduction, with an ovoid section, showing in light grey the remains of the scars from the previous stages that sought to reduce the thickness of the piece; in dark grey the negatives produced by formatting the body of the piece are shown, and in light pink the negatives from formatting the edges and contour of the piece. **B** The reverse of the piece and the shallow depth of the negatives produced by percussion are shown. Reference scale: 6 cm

The next stage continues the process until the expected thickness is reached while the lateral body and edges are reduced by the continuous alternating technique on both sides (Fig. 3C-D). This generates smaller negatives (± 3 cm long by 1 cm wide) due to the greater control of the direction and force applied, generating invasive negatives on the surface of the previous negatives and riding the protruding edges of the previous scars. Here more scars are generated per round, about 22 per side (Fig. 4).

**Fig. 5:**
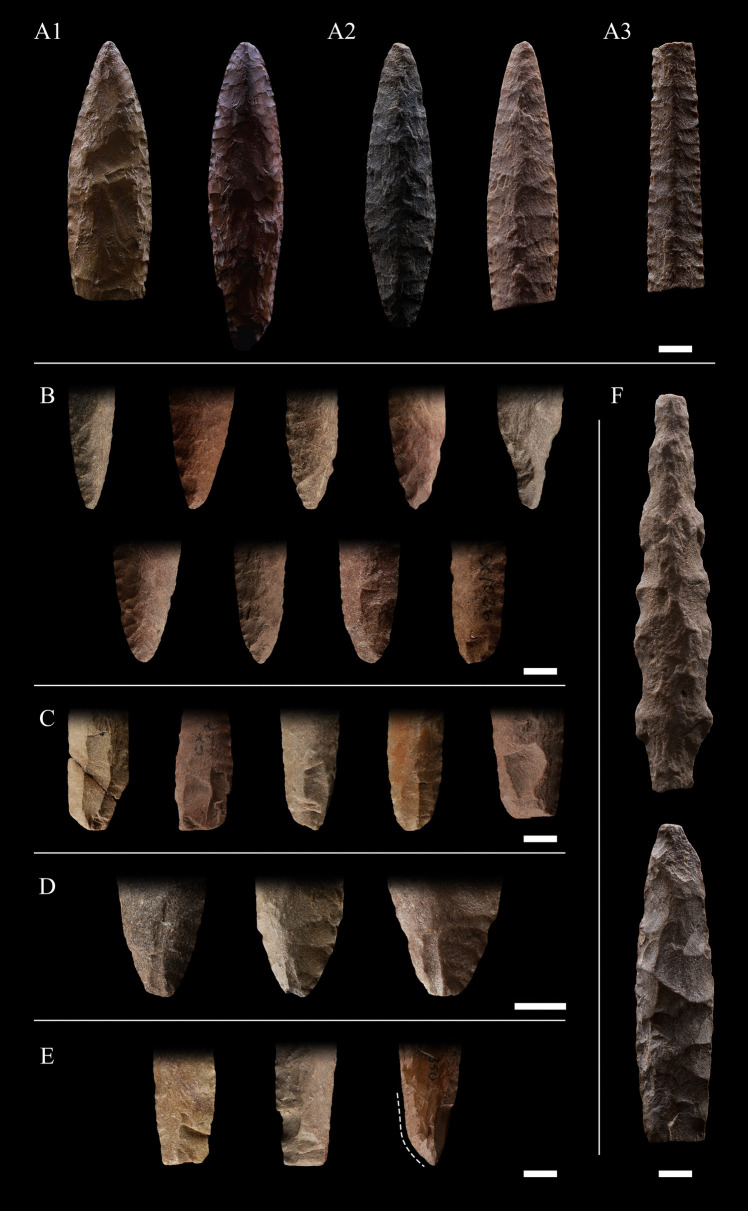
Shaping flaking patterns, bases variations, techniques and preforms identified in El Jobo projectile points sample. **A1)** Irregular flaking pattern. **A2**) Collateral flaking pattern. **A3**) Parallel flaking pattern.** B**) Shape variations of bases.** C**) Fluting technique.** D**) Fluting-like scars.** E**) Basal reconfigurations** F**) Preforms. Reference scale: 1 cm

For the next stage of bifacial reduction, the piece (preform) should be about 3 cm wide and the thickness would already be below 1 cm (Fig. 5F). This next stage is developed with a retouching tool, and the edges are pressure-formed, reducing them in a continuous alternating manner, until the width of the piece is almost double its thickness. The edges are shown with obtuse angles of up to 45 degrees and the edges, in most cases, are shown in the form of a “zigzag.” The landscape of the faces of the piece only reveals fragments of the scars of the second reduction stage on the central rib of the piece. The multiple negatives of the third reduction stage can be seen by dozens and superimposed on the intermediate body, and already on the edges, the negatives generated by pressure, up to 2 mm long, can be preserved continuously on both faces of the piece. The completion of the point is achieved through the elaboration of the bases, if any, and the retouching of the edges. This process is also conducted in a continuous alternating manner through controlled percussion or pressure flaking (Fig. 5; Jaimes 1999, 2005).

No major differences are hypothesized between the techniques applied for *Jobo 1*, *Jobo 2*, *Jobo 3* and *Jobo-Clovis*, which presented both collateral and irregular (random) flaking patterns of the shaping phase (Fig. 5A1-2). The retouch of the edges can exhibit a parallel pattern, best observed at the extremes of the projectile points, and clearly noted in the *Jobo-Eden* Type (Fig. 5A-3). In the case of the *Jobo-Serrado*, the"saw teeth"alternate on each side of the blade.

### Qualitative description

**Fig. 6:**
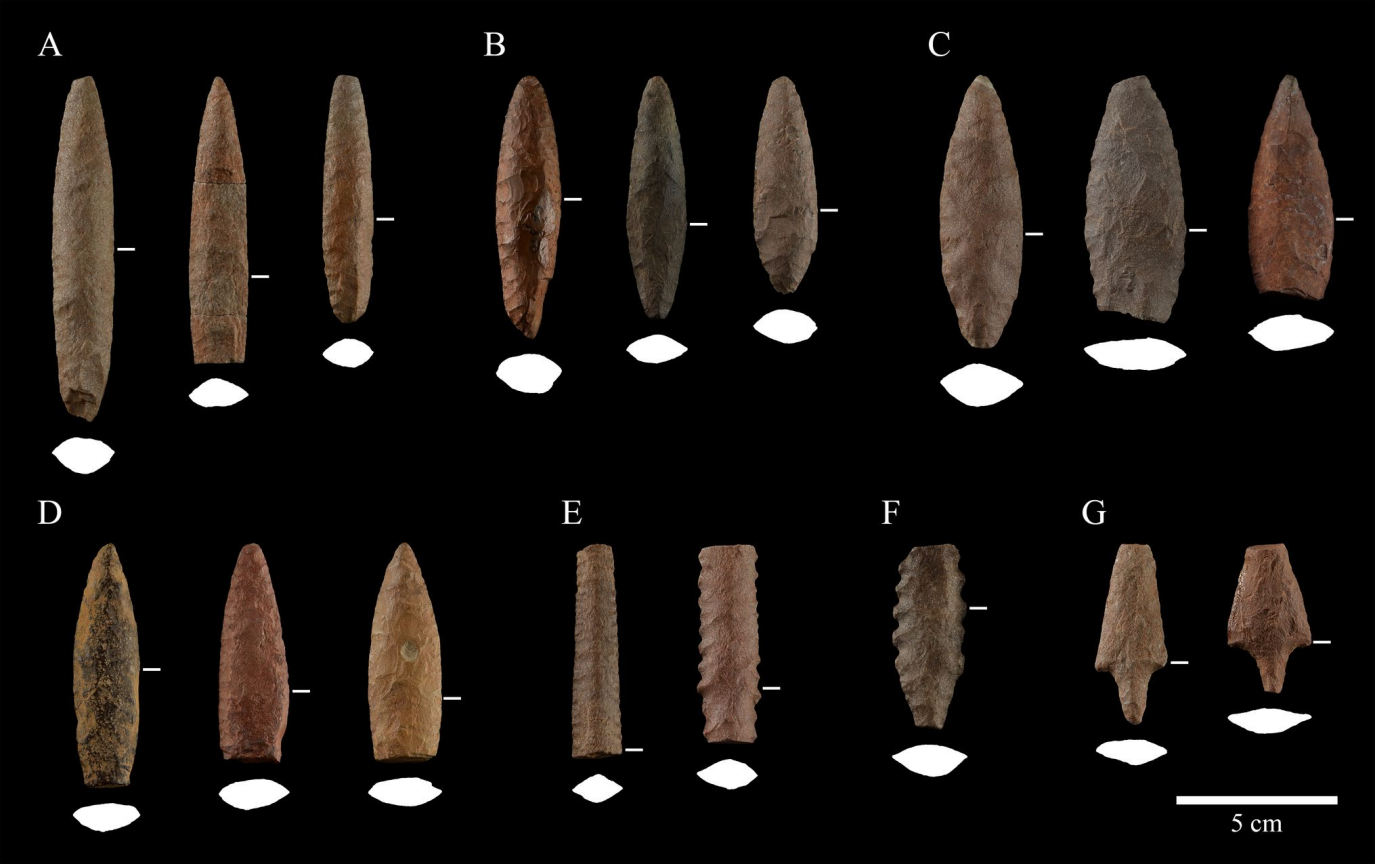
El Jobo projectile point samples and preliminary classification. The cross-sections were obtained from the widest portion of the projectile point. **A**) Jobo 1. **B**) Jobo 2. **C**) *Jobo 3*. **D**) *Jobo-Clovis*. **E**) *Jobo-Eden*. **F**) *Jobo-Serrado*. **G**) *Las Casitas*

The attributes observed in our sample set are consistent with former descriptions of El Jobo projectile points (Admiraal 2013; Cruxent and Rouse 1958; Jaimes 1999; Jaimes et al. 2024a). Considering the whole sample (i.e. = 116), a preliminary classification of six groups of forms was established: Clovis-like (*Jobo-Clovis*), Eden-like (*Jobo-Eden*), and serrated (*Jobo-Serrado*) points, as previously identified by Jaimes et al. (2024a), and three main groups that divide the most representative forms in the sample, defined in the present work as Types 1 (*Jobo 1*), 2 (*Jobo 2*), and 3 (*Jobo 3*) (Fig. 6). Additionally, a small sample of points from the Las Casitas tradition (Arroyo et al. 1971) was identified.

*Jobo 1* (n = 25) may be considered as the more conservative type in terms of the initial descriptions of El Jobo (Admiraal 2013, p. 13). We define this type as thick-bodied fusiform points with biconvex mid-section. Jobo 1 is also distinguished by its relative great length and narrow body. The base of this Type can be either pointed, almost symmetrical with the distal part, or rounded (Fig. 6A). Reductions and probably lateral reconfiguration of the base by fracture or flaking in conjunction with fluting were also observed.

*Jobo 2* (n = 32) is considered as short lanceolate bi-points, or with a shorter base, *i.e.*, with the maximum width near the proximal part of the point. The mid-section is biconvex, and the bases can be pointed, rounded, or stemmed. Basal reconfiguration, transverse basal reduction, lateral reduction, flaking and micro-flaking, fluting, and fluting-like scars (less than 5 mm of width) have been observed (Fig. 6B). Here, we define micro-flaking as a narrow one (less than 5 mm); elongated flaking scars are located at the base of the point.

*Jobo 3* (n = 30) is defined by its foliate (leaf-shaped) or ovoid shape. The mid-section of these objects is wide and biconvex, while the bases are either pointed or rounded and may exhibit fluting and fluting-like scars.

In the case of the *Jobo-Serrado* (n = 3) Type, only fragmented points have been identified in the collection. However, these fragments and other published points (see Jaimes et al. 2024a: figs. 6L, 7H) can exhibit any of the shapes previously described for the preceding types, but with serrated edges and a stem base.

**Fig. 7:**
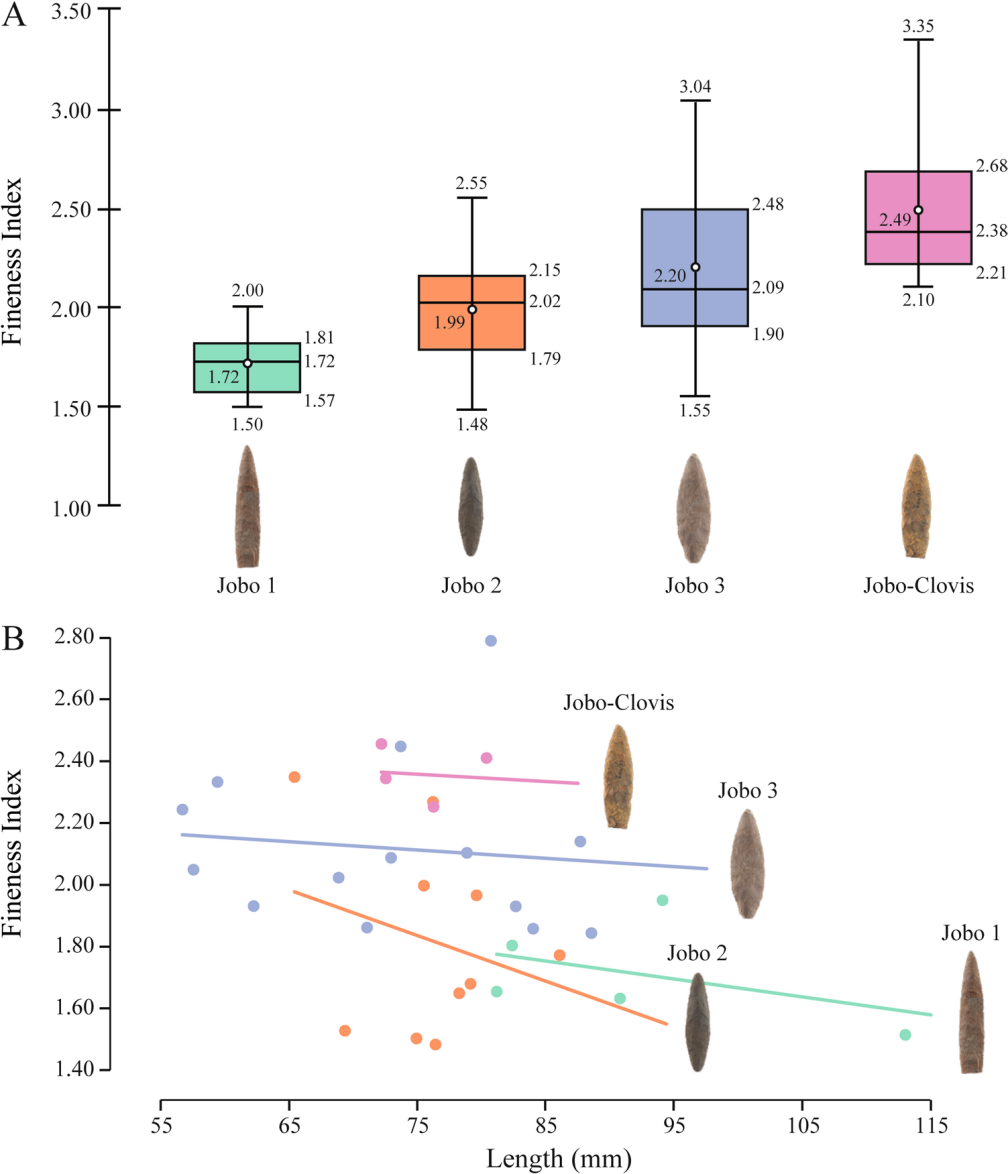
**A**) Variation in fineness index within each El Jobo Type. Statistical values within each Type are shown. From bottom to top: minimum value, lower quartile, median, upper quartile, maximum. The value in the box is associated with the white point corresponding to the mean. **B**) Fineness index according to maximum length with slopes for each El Jobo Type using only complete points

The classification of the *Jobo-Clovis* (n = 6) and *Jobo-Eden* (n = 2) types, first proposed by Jaimes et al. (2024a), is based on similarities in morphological features and techniques of edge retouching with known technologies from North America. Jobo-Clovis exhibits a distinctive lanceolate shape, showing a wide biconvex midsection and an apparent straight base. It presents scars from overshot flakes, characteristic of the North American Clovis tradition. The Jobo-Eden is the type with a diamond-shaped medial section. Jobo-Eden presents edge formatting features, with oblique parallel pressure retouching, similar to the Scottbluff-Eden specimens (Knell 2007).

The final group of points identified is known as the *Las Casitas* (n = 5) tradition (Arroyo et al. 1971), which exhibits distinct morphological characteristics when compared to El Jobo. These small-sized points are distinguished by a triangular body with an ovate stem, straight to obtuse shoulders, straight edges, an elliptical body section, and a round stem section. Although not included in the morphometric analysis, this kind of point was first described as a typology associated with El Jobo points, one that can be derived from the production sequence (Cruxent and Rouse 1958) and as such we provide documentation of it. The overall proportions, including the stem of the form, are quite different from the other El Jobo points.

Another group of points (n = 13) could not be identified within any of these Types or tradition due to their high fragmentation, unique individual morphology, or because they are preforms. Their measurements are also included in the supplementary material.

While the characteristics mentioned above differentiate the overall shapes of the projectile points, the measurements taken reveal overlapping and homogeneous values. However, some discernible trends can be identified when comparing the Types with each other, as exemplified by the width and the length of the complete samples (Table 1, Supplementary material 2).

**Table 1:** General measurements of primary El Jobo types

Type	Min	Q1	Q2	Q3	Max
*Jobo 1* (*n* = 25)					
Width (mm)	15.83	17.45	17.95	19.41	20.43
Thickness (mm)	9.45	9.81	10.46	11.57	13.28
Fineness index	1.5	1.57	1.72	1.81	2
Length* (mm)	81.2	81.81	90.82	103.6	113.06
*Jobo 2* (*n* = 32)					
Width (mm)	16.93	19.17	20.33	22.49	24.75
Thickness (mm)	8.34	10.05	10.62	11.44	12.94
Fineness index	1.48	1.75	1.98	2.11	2.55
Length* (mm)	61.2	69.38	76.24	79.17	86.12
*Jobo 3* (*n* = 30)					
Width (mm)	15.73	20.48	22.78	24.64	27.63
Thickness (mm)	7.42	9.3	10.43	11.5	14.47
Fineness index	1.55	1.93	2.12	2.46	3.04
Length* (mm)	56.7	61.55	73.34	83.04	88.58
*Jobo-Clovis* (*n* = 6)					
Width (mm)	18.01	19.86	22	25.57	31.85
Thickness (mm)	8.56	8.88	9.3	9.54	9.56
Fineness index	2.1	2.21	2.38	2.68	3.35
Length* (mm)	72.1	72.3	74.42	79.37	80.41

*Q* Quartile

*Only of complete points

Different fineness indeces were also identified (Fig. 7A). Although an overlap is observed, a progressive increase of the median (Q2) and its max value can be identified if Types are arranged from *Jobo 1* to *Jobo-Clovis*. This increase can be primarily attributed to the width of the samples, as the thicknesses, except for *Jobo-Clovis*, do not differ substantially between Types (Table 1). Additionally, for each Type, we detected that an increase of the length of the points is accompanied with a decrease of the fineness index of the medial sections, according to the obtained slopes (Fig. 7B).

Las Casitas tradition is a distinct manufacture process, similar to the reduction sequence scheme described by de Sousa and Okumura (2020) for Rioclarense points. The examined points exhibit trespassed and selective un-trespassed negatives organization (Table 2).

**Table 2:** Main technological attributes in El Jobo types and Las Casitas tradition

Type	Medial section	Max. width location	Flaking pattern	Negative organization	Retouch pattern	Other
*Jobo 1* (*n* = 25)						
	Biconvex (100%)	Medial (60%)	Collateral-Irregular (32%)	n/a	Continuous alternate (100%)	Fluting
		Proximal (36%)	Collateral (28%)			Basal reconfiguration
*Jobo 2* (*n* = 37)						
	Biconvex (100%)	Medial (54%)	Collateral-Irregular (49%)	n/a	Continuous alternate (100%)	Fluting
		Proximal (43%)	Irregular (35%)			Fluting-like scars
*Jobo 3* (*n* = 24)						
	Biconvex (92%)	Proximal (58%)	Irregular (50%)	n/a	Continuous alternate (100%)	Fluting
	Plan-convex (8%)	Medial (38%)	Collateral (21%)			Basal reconfiguration
*Jobo-Clovis* (*n* = 6)						
	Biconvex (100%)	Medial (67%)	Collateral-Irregular (83%)	n/a	Continuous alternate (83%)	Basal reconfiguration
		Distal (17%)	Not visible (17%)		Not visible (17%)	
*Jobo-Serrado (n* = *3)*						
	Biconvex (100%)	Proximal (66%)	Collateral (33%)	n/a	Continuous alternate (67%)	Serration
		Medial (33%)	Collateral-Irregular (33%)		Not visible (33%)	
*Jobo-Eden (n* = *2)*						
	Lozenge (100%)	n/a	Parallel (100%)	n/a	Continuous alternate (100%)	n/a
*Las Casitas (n* = *5)*						
	Biconvex (100%)	n/a	n/a	Selective trespassed(80%)	Continuous alternate (80%)	n/a
				Selective untrespassed (20%)	Continuous normal (20%)	

### Whole-outline geometric morphometric analysis

As previously stated, for the geometric morphometric analysis, only the complete points from our sample were used, which only encompasses some of the points from *Jobo 1*, *Jobo 2*, *Jobo 3*, and *Jobo-Clovis*. The *Jobo-Eden* and *Jobo-Serrado* samples yielded only fragmented points, while the *Las Casitas* sample was excluded due to its markedly different morphology.

**Fig. 8:**
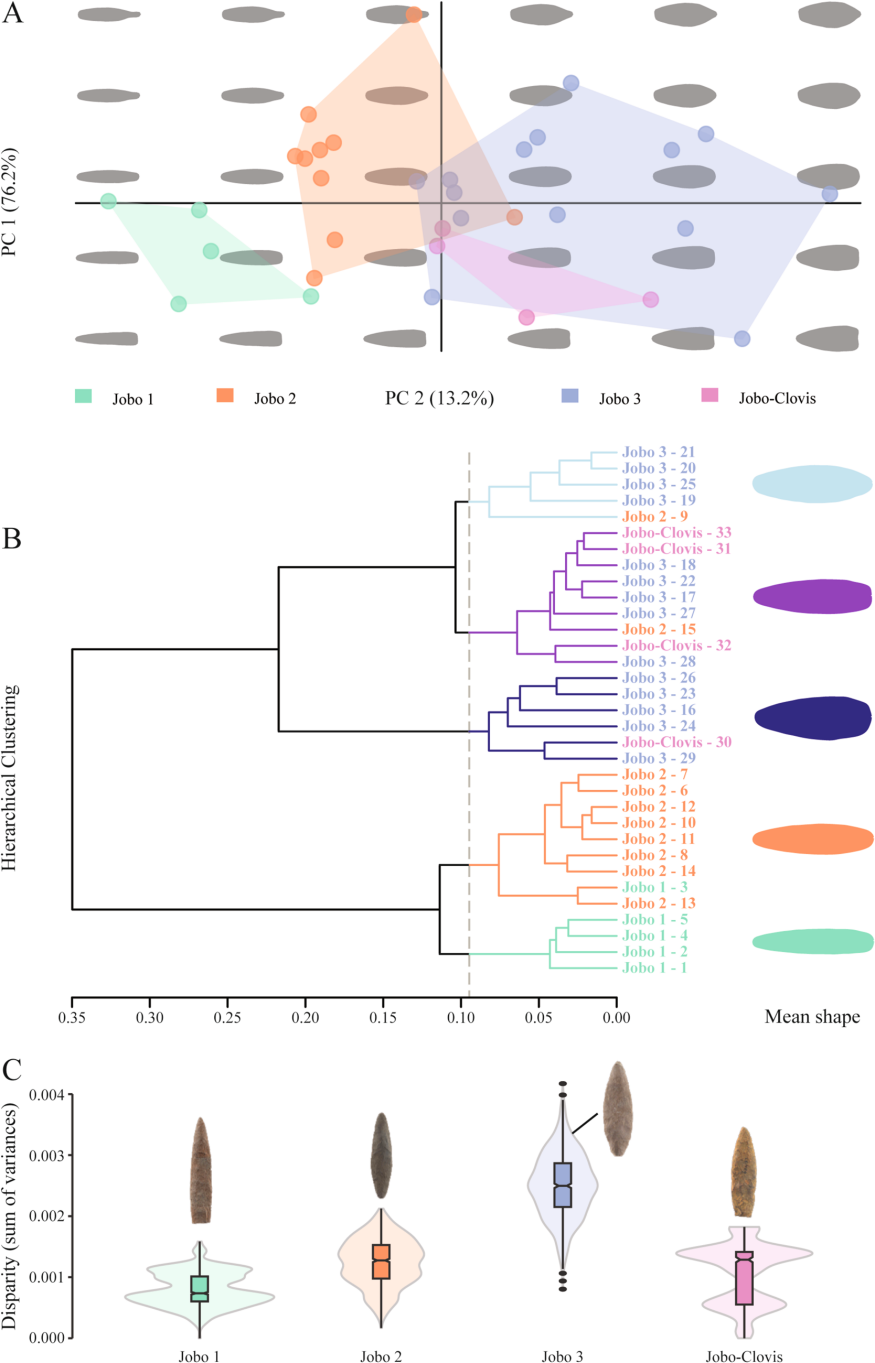
Whole-outline geometric morphometrics for the complete set (n = 33) of El Jobo projectiles. **A**. PCA scatterplot of the two first axis. Colors follow the preliminary classification for each Types. The morphospace includes the shape variation represented by the two first axes. **B**. Dendrogram from hierarchical clustering and mean shapes derived from the five main clusters (k = 5). Name colors follow Types, while branch and shape colors follow the dendrogram. **C**. Violin plots showing the disparity expressed as the sum of variance for each of the Type (see Supplementary material 4 for statistics). The same color code is used as for A

The morphospace resulting from the first two PC axes represents 89.4% of the total shape variance (Fig. 8). PC1 (76.2%) is mainly driven by the width of projectile points, ranging from lanceolate/fusiform to ovoid shapes (Fig. 8). In contrast, PC2 (13.2%) shows variations in the distal and proximal parts (Fig. 8), suggesting that shape variation expressed within this axis is sensitive to the deformations produced by the base shaping, reconfigurations and fractures. Three main clusters can be distinguished on PC1: one with *Jobo 1* points, another with *Jobo 2* points, and the last with *Jobo 3* and *Jobo-Clovis* points (Fig. 8). A small overlap is detectable in the morphospace with a *Jobo 2* sample falling into the cluster defined by *Jobo 3* and *Jobo-Clovis*.

The dendrogram derived from the hierarchical clustering (HC) reveals at least two large clusters in line with the distribution from the morphospace (Fig. 8). Approaching lower dissimilarities, the HC reveals at least the cluster defined by *Jobo 1*, *Jobo 2* and the last by *Jobo 3* with the *Jobo-Clovis*, itself divided into three potential clusters (Fig. 8). Comparison of the mean shapes by HC cluster also shows that the distribution in the dendrogram is mainly driven by the width of the projectile points (Fig. 8).

Furthermore, projectile points responsible for the proximity between Types and the overlap observed in the PCA and HC exhibited a unique morphology. These projectile points were the only ones in the sample with a stemmed base ("0015") and a reconfigured base ("0720"and"0786"), leading to presume that they generate a biased closeness with the points showing fragmented bases. To address this issue, the same methodology was applied to the sample without these unique projectile points, resulting in more distinct groupings in both the PCA and HC (Supplementary material 3). Thus, the fragmentation of the projectile points can significantly impact the observed variations, particularly when the sample size is limited, and the shapes are relatively similar (see Supplementary material 3).

A comparison of shape disparity between the four Types reveals that *Jobo 3* shows a greater disparity, while the other three Types show relatively equivalent ranges of shape disparity. *Jobo 3* is the best sampled and this result might be due to a sampling effect. Nevertheless, the Wilcoxon test statistically supports the difference between each of the Types (p-value < 0.05; see Supplementary material 4), even between *Jobo 3* and *Jobo-Clovis*, confirming the clusters found in PCA and HC.

### Geochemical analysis

With only one exception, all investigated samples have high silicon concentrations (33–48%; Table 3; Supplementary material 5). This, together with the surface structure of the samples and how the rocks broke during tool production, lead to the classification as silicate rocks, mainly siltstones and sandstones. This translates into more than 70% SiO_2_ for most samples, which classifies the samples as quartz-and silicate-rich. The silicate rocks were affected by weak to moderate metamorphosis, i.e., the rocks are quarzitic silt and sandstones, where the metamorphosis led to more compaction of the rocks that made them harder and with more cementing of minerals. The variability of cementing minerals is indicated by the various concentration of other elements such as Ca, K, Cl, Fe or Mg, which can reach sometimes 1% or even more than 10% for selected samples. This indicates the variability of the mineral assemblages, apart from the high Si concentration. Likely, on top of quartz, there are layered silicates abundant that are formed during metamorphosis by percolating solutions. However, it remains unclear, to which extent the variability of the elemental composition represents the diversity of the sedimentary conditions and/or the overprint during metamorphosis.

**Table 3:** Elemental composition of major elements found in all points. Values are given as mean values and standard error of the mean (SEM)

	number of points	Al	Ba	Ca	Cl	Cr	Fe	K	Mg	S	Si	Ti	V
		mean ± SEM [%]	mean ± SEM [%]	mean ± SEM [%]	mean ± SEM [%]	mean ± SEM [%]	mean ± SEM [%]	mean ± SEM [%]	mean ± SEM [%]	mean ± SEM [%]	mean ± SEM [%]	mean ± SEM [%]	mean ± SEM [%]
Jobo1	8	3.44 ± 0.39	0.009 ± 0.002	0.23 ± 0.09	3.40 ± 0.71	0.0030 ± 0.0006	0.25 ± 0.04	0.23 ± 0.03	0.65 ± 0.07	0.11 ± 0.03	43.3 ± 1.3	0.09 ± 0.01	0.0018 ± 0.0002
Jobo2	10	4.50 ± 0.89	0.011 ± 0.001	0.42 ± 0.22	3.23 ± 0.65	0.0044 ± 0.0004	0.87 ± 0.35	0.32 ± 0.08	0.73 ± 0.10	0.18 ± 0.05	42.0 ± 1.9	0.14 ± 0.03	0.0026 ± 0.0006
Jobo3	16	3.63 ± 0.44	0.014 ± 0.002	0.18 ± 0.07	2.58 ± 0.44	0.0044 ± 0.0005	1.00 ± 0.56	0.23 ± 0.02	0.72 ± 0.07	0.22 ± 0.03	44.6 ± 0.7	0.10 ± 0.02	0.0024 ± 0.0005
JoboAserrado	2	2.81 ± 0.02	0.012 ± 0.000	0.07 ± 0.03	3.78 ± 2.18	0.0028 ± 0.0001	0.19 ± 0.04	0.18 ± 0.00	0.67 ± 0.07	0.11 ± 0.05	44.2 ± 2.4	0.08 ± 0.01	0.0010 ± 0.0000
JoboClovis	5	6.18 ± 1.60	0.032 ± 0.014	3.72 ± 3.38	3.71 ± 1.67	0.0042 ± 0.0006	5.51 ± 2.54	0.49 ± 0.08	0.86 ± 0.26	0.32 ± 0.07	36.1 ± 4.0	0.13 ± 0.02	0.0055 ± 0.0017
JoboEden	1	2.74	0.01	0.01	4.46	0.0042	0.29	0.21	0.65	0.10	45.5	0.13	0.0015
LasCasitas	3	2.98 ± 0.80	0.007 ± 0.001	0.38 ± 0.25	4.19 ± 2.95	0.0035 ± 0.0004	0.19 ± 0.08	0.21 ± 0.01	0.79 ± 0.32	0.18 ± 0.10	43.4 ± 2.2	0.05 ± 0.01	0.001 ± 0.0003
Preform	1	2.38	0.01	0.14	2.61	0.0018	0.13	0.15	0.51	0.22	42.4	0.04	0.0017
Unidentif	4	4.09 ± 1.66	0.012 ± 0.011	0.30 ± 0.31	1.83 ± 1.59	0.0041 ± 0.0007	0.24 ± 0.18	0.24 ± 0.12	0.74 ± 0.15	0.17 ± 0.20	44.9 ± 3.8	0.06 ± 0.03	0.0014 ± 0.0007
mean	50	3.99 ± 0.31	0.014 ± 0.002	0.61 ± 0.36	3.08 ± 0.33	0.0040 ± 0.0002	1.15 ± 0.38	0.27 ± 0.02	0.72 ± 0.04	0.19 ± 0.02	42.9 ± 0.7	0.10 ± 0.01	0.0024 ± 0.0003

Sample “1012” is the only sample with exceptional high calcium concentration, which could translate into 43% CaCO_3_, if it is a carbonatic rock (Supplementary material 5). This is likely as the silicon concentration is low at the same time. However, also carbonatic rocks can be formed in an evaporative setting like a shallow marine environment. As carbonate rocks are typically rather weak, also this material likely underwent metamorphosis, and the high iron concentration may argue that here some Fe/Ca carbonates are included, improving the hardness of the rocks. Although this sample yields a high Ca and Fe concentration, magnetic susceptibility did not show any result and low Mg concentrations do not argue for the formation of dolomite during metamorphosis as often observed for carbonatic rocks. Overall, 26% of the samples showed a positive response during magnetic susceptibility measurements.

Some samples contain specifical elemental composition such as high aluminum (samples “0001”, “0022”, “0074”) and thus more aluminium-silicates. Samples “0003” and “0021” contain much chlorine, arguing for the formation of large amounts of chloride-bearing minerals during metamorphosis; samples seem to be either shist/chert and have been formed from clay stones, eventually. Elemental concentration of Fe (iron) is very high especially in samples “0348” and “1012” (Supplementary material 5). Specifically, the trace element composition (e.g., Ni, Mn, Cu, and As) may highlight some differences between the sample Types. They are abundant in most samples of *Jobo-Clovis* Type and only one additional sample from *Jobo 3*, but absent in this combination for most other samples.

## Discussion

The data gathered on the manufacturing process supports a homogeneous shaping pattern of the points through almost all the reduction sequence, which suggests the maintenance of a stable tradition. The width variation observed among the Types can be attributed to the decisions made during the final stage of reduction, prior to retouching. Based on the high amount of irregular to collateral and irregular flaking patterns, we hypothesize that in the shaping phase, the artisan's objective was to control the rock irregularities while achieving the appropriate dimensions of the point rather than maintaining a symmetrical distribution of the flaking negatives as observed in Clovis and other traditions of North America (Sholts et al. 2012). Nevertheless, the intention to maintain a symmetry through a parallel pattern is observed in the retouch of the edges, especially in some of the distal or proximal parts of the points.

The results of the qualitative descriptions and measurements of El Jobo points show overlapping values and different trends related to the width and length of the proposed typologies. A progressive increase in the fineness index of the points is proposed; and the observed relation between the fineness index and lengths can be interpreted as a physical constraint, implying that an increase in the size of the points requires an increase in thickness to support their structure, thus reducing the ratio in relation to length.

A technological diversity was detected in the manufacture of the different types of bases. We hypothesize that this is an indicator not only of the variety in the design of point hafting, but also a differentiation in functionalities for a same morphology (see Howard 1996; Mayer-Oakes 1986: fig. 11). Differences in lengths (between 5 and 11 cm), weights (from 12 to 35 g), shapes, and hafting techniques may reflect a great diversity of functions adaptable to different hunting strategies or prey sizes, yet to be subject of further investigation (see Prates et al. 2022).

The morphometric analyses and the qualitative classifications converge in the recognition of different point types, even if morphologically close. Width variation was the main distinction among a continuum of similar shapes. The Types identified by the morphometric analyses are close in shape, but Types 1, 2 and the combination of Type 3 and Clovis-like Type are all significantly different.

Our elemental analysis of the samples shows the presence (with one exception) of exclusively quarzitic rocks, exhibiting some variation in elemental composition, perhaps reflecting diverse sources of the sedimentary rocks. The Jobo-Clovis type exhibited a common and distinctive elemental composition, different from the other types – with one sample of Jobo 3 exhibiting a similar composition. This may indicate a common and singular source for the Jobo-Clovis Type points studied. It is noteworthy the prevalence of marine sedimentary rocks that underwent metamorphosis during orogenesis in the Falcon Basin (Albert-Villanueva et al. 2017). This resulted in a widespread distribution of quarzitic rocks, which, due to their structure and hardness, are well-suited for tool production (Nami 1994, 1999).

Following previous hypothesis (see Jaimes et al. 2024a), the available rock types may have influenced point design. Fine-grained materials, as chert, likely supported the precise flaking needed for Clovis lanceolate shapes. In contrast, coarser materials such as sandstone may not have merely favored broader Jobo-like points. The latter may have fundamentally constrained and defined that technological choice, as their fracture properties limited the feasibility of producing more refined shapes.

The magnetic properties of some of the projectile points can be attributed to primary minerals like hematite and magnetite present in the rocks (Hambach et al. 2008); however, this seems less likely for our samples, which either contain magnetic particles or do not. A more probable explanation is the formation of magnetic minerals during heating in (camp) fires (Hambach et al. 2008; Zeeden et al. 2015) for purposes such as cleaning, hardening, or improving the fracture properties of the projectile points (Nami et al. 2000; Schmidt 2020).

These interpretations—regarding the influence of raw material and magnetic properties of the artifacts —remain speculative until integrated studies, using e.g., experimental archaeology and further mineralogical characterization (Brown et al. 2009) are conducted.

**Fig. 9:**
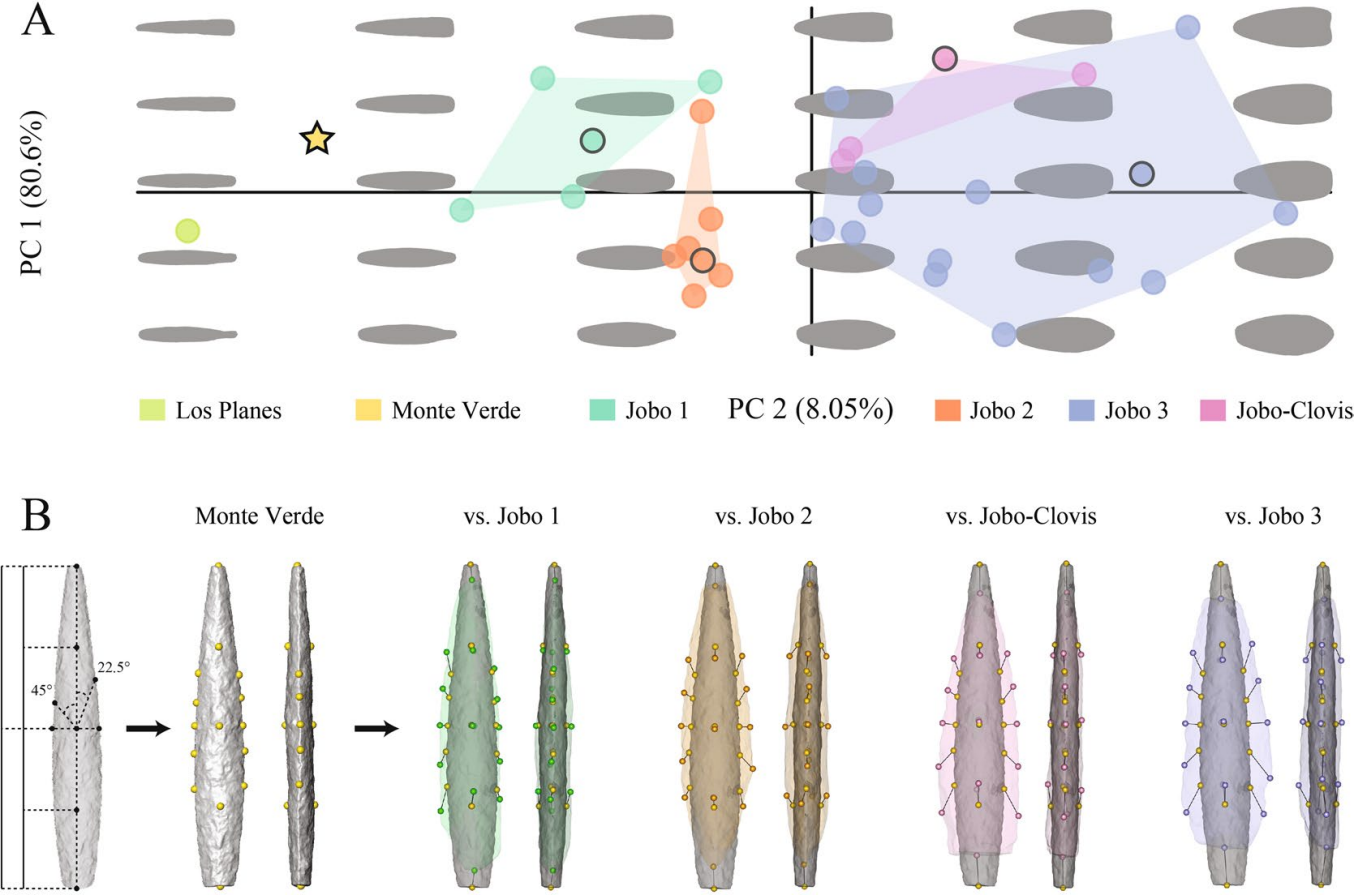
Shape similarities between the studied projectile points in Venezuela and one Monte Verde point (Dillehay et al. 2019). **A.** Re-run of the analysis in Fig. 8 including a Monte Verde and a Los Planes projectile point (without the three potentially base-biased Jobo arrow points, see results and Supplementary material 3). The black outlines indicate the samples selected for detailed visualization. **B.** Detailed shape comparison between the Monte Verde projectile point and selected Jobo points. A diagram shows the quantification procedure with 3D landmarks of type 3

The results of the morphometric analysis including the Monte Verde projectile point show an intermediate position of the Monte Verde point between the *Jobo 1* arrow points and the Los Planes point; there is a strong similarity in shape between the Monte Verde arrow point and the Jobo 1 (Fig. 9).

In order to elucidate the morphological, technological, morphometric and geochemical variability that has been observed, we put forward three potential explanations for this diversity: (1) A likely late interrelation (north-to-south) within northern technologies (e.g. Clovis), which would explain the progressive increase in the width of the projectile points as well as the similitudes between *Jobo 3* and *Jobo-Clovis* Types; (2) the independent developments in the Lara-Falcon region resulting in convergent techniques and shapes; or (3) a South American technological innovation that was subsequently incorporated into northern traditions (south-to-north). Alternatively, multidirectional and long-term interactions across the continent could have contributed to this complex pattern of variations (Admiraal 2013). Moreover, the similarities with the Monte Verde II projectile points, the El Jobo fragments from Panama, and the potential relationships with the thick-bodied lanceolate projectile points from North America reinforce the hypothesis of a widespread technological development of an El Jobo-like tradition, which remains to be studied. Consequently, our third explanatory hypothesis should be tested. The discovery in Belize of a biface tool technological complex and comparisons with tools in South America and Mesoamerica has led to suggestions of cultural transmission across large geographic areas (Prufer et al. 2019).

Lastly, one can assume that the fusiform shape of El Jobo projectile points were designed for penetration. The extent to which these projectiles represent a distinctive ballistic innovation could be tested in comparative studies of design (Friis-Hansen’s 1990; Sitton et al. 2020; Grady and Churchill 2023) and of penetration potential (Buchanan et al. 2022; Eren et al. 2022).

## Conclusions

The northwestern region of Venezuela was occupied by human groups who developed and diversified a bifacial reduction lithic technology (Bryan and Gruhn 2003). The El Jobo fusiform projectiles represent a distinctive technological innovation used through at least three millennia, whose use is evidenced by their presence in two of the most notable megafaunal hunting sites on the continent (Bampi et al. 2022; Jaimes et al. 2024b).

The results of this work reveal a greater diversity than that previously documented, thereby broadening perspectives on technological adaptations and relationships during the late Pleistocene. As proposed, late Pleistocene populations in northwestern Venezuela achieved control and knowledge of the diverse raw materials in the region, which they adapted to the different tools developed (Jaimes et al. 2024a). Furthermore, the appearance of fluting, fluting-like scars and a wide range of basal shapes at El Jobo indicates the use of diverse types of haftings and potential functional diversity of the points.

The continuity in the morphospace, the morphological and technological diversity, as well as the relationships identified with other technologies (Jaimes et al. 2024a) are interpreted by us as reflecting an exchange of technologies across large geographic areas of the Americas that may be causing the variation within El Jobo. Further testing of this hypothesis is required with expanded temporal, stratigraphical and geographical information beyond that reported in this paper.

## Supplementary Information

Below is the link to the electronic supplementary material.

Supplementary file1 Dates of sites with the presence of El Jobo projectile points. Calibration of all available radiocarbon dates associated with El Jobo technology, using the OxCal v4.4.4 software and IntCal20 calibration curves (Bronk Ramsey, 2021; Reimer et al., 2020) (XLSX 13 KB)

Supplementary file2 El Jobo projectile points database (XLSX 29 KB)

ESM 3 Whole-outline geometric morphometrics for the reduced set of Jobo projectiles. A. PCA scatterplot of the two first axis. Colors follow the preliminary classification for each Types. The morphospace includes the shape variation represented by the two first axes. B. Dendrogram from hierarchical clustering and mean shapes derived from the five main clusters (k=5). Name colors follow Types, while branch and shape colors follow the dendrogram. C. Boxplots showing the disparity expressed as the sum of variance for each of the Type (see Supplementary material 4 for statistics). The same color code is used as for A (PNG 508 KB)

High Resolution Image (TIF 2.85 MB)

Supplementary file4 Pairwise Wilcoxon signed-rank test with Bonferroni correction including all Types for both the complete and reduced dataset. Lower triangle = W statistics; Upper triangle = p-values (XLSX 10 KB)

Supplementary file5 Elemental composition in all points (XLSX 61 KB)

## Data Availability

Data generated and analyzed in this study are provided in this article and Supplementary Materials, additional details are available on request through the corresponding author. The sample used for the geometric morphometric analysis (i.e., n = 33), were scanned using an Artec Space Spider equipment, and consequently 3D-reconstructed using Artec Studio 17 Professional software. Following these acquisitions, we created an open access collection of the 3D models for sharing and further evaluations, available on https://www.morphosource.org/projects/000670116.
