# Supplementary figures and images for: A morphological, morphometric and geochemical characterization of the El Jobo projectile points – diversity and significance in early human populations across the Americas
Source: Archaeol Anthropol Sci. 2025 Aug 16;17(9):184. doi: 10.1007/s12520-025-02296-2 (PMC12357808; doi:10.1007/s12520-025-02296-2)

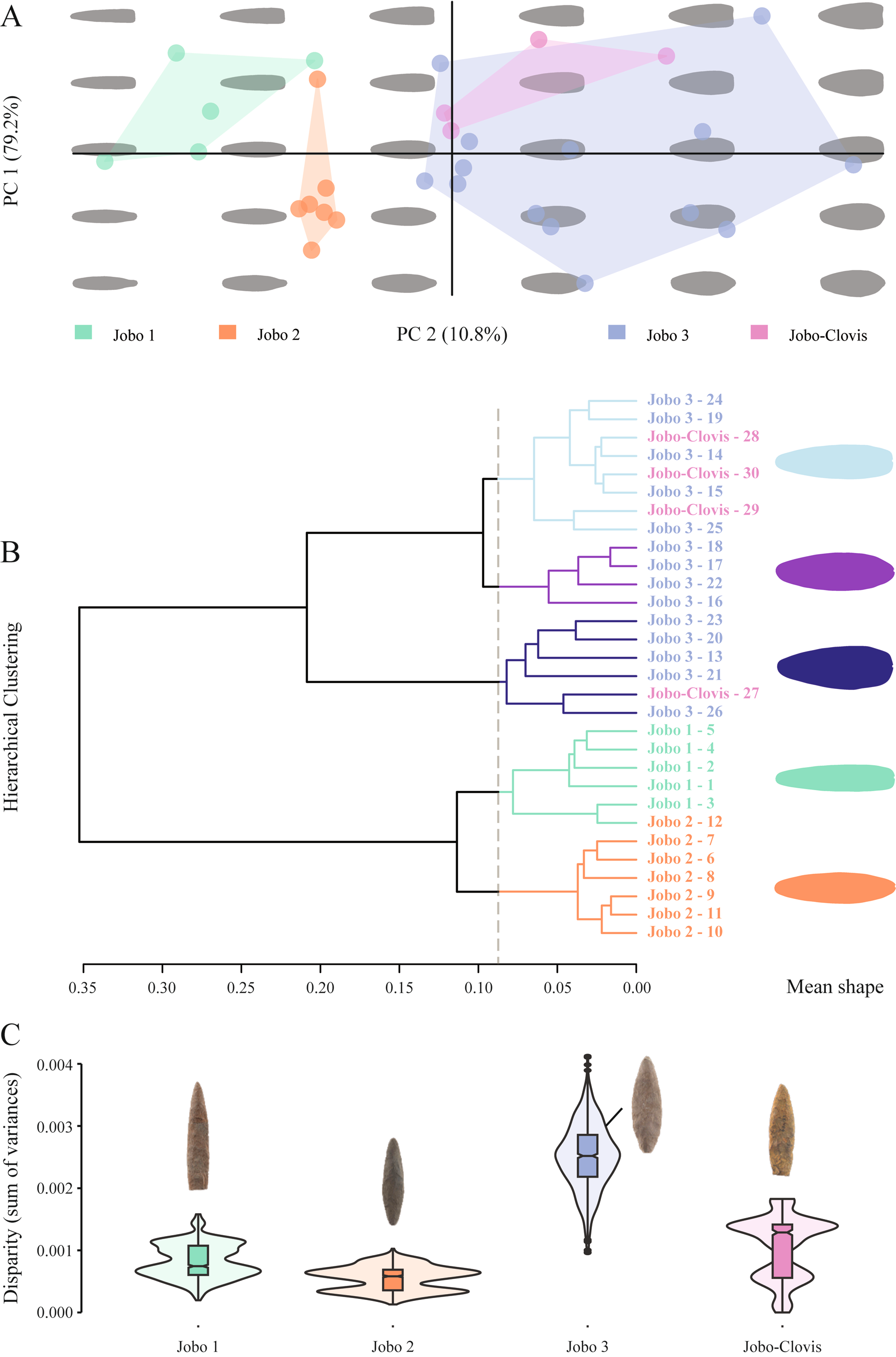

Supplement: Supplementary file 3 — Whole-outline geometric morphometrics for the reduced set of Jobo projectiles. A. PCA scatterplot of the two first axis. Colors follow the preliminary classification for each Types. The morphospace includes the shape variation represented by the two first axes. B. Dendrogram from hierarchical clustering and mean shapes derived from the five main clusters (k=5). Name colors follow Types, while branch and shape colors follow the dendrogram. C. Boxplots showing the disparity expressed as the sum of variance for each of the Type (see Supplementary material 4 for statistics). The same color code is used as for A (PNG 508 KB) [file 12520_2025_2296_Fig10_ESM.png]

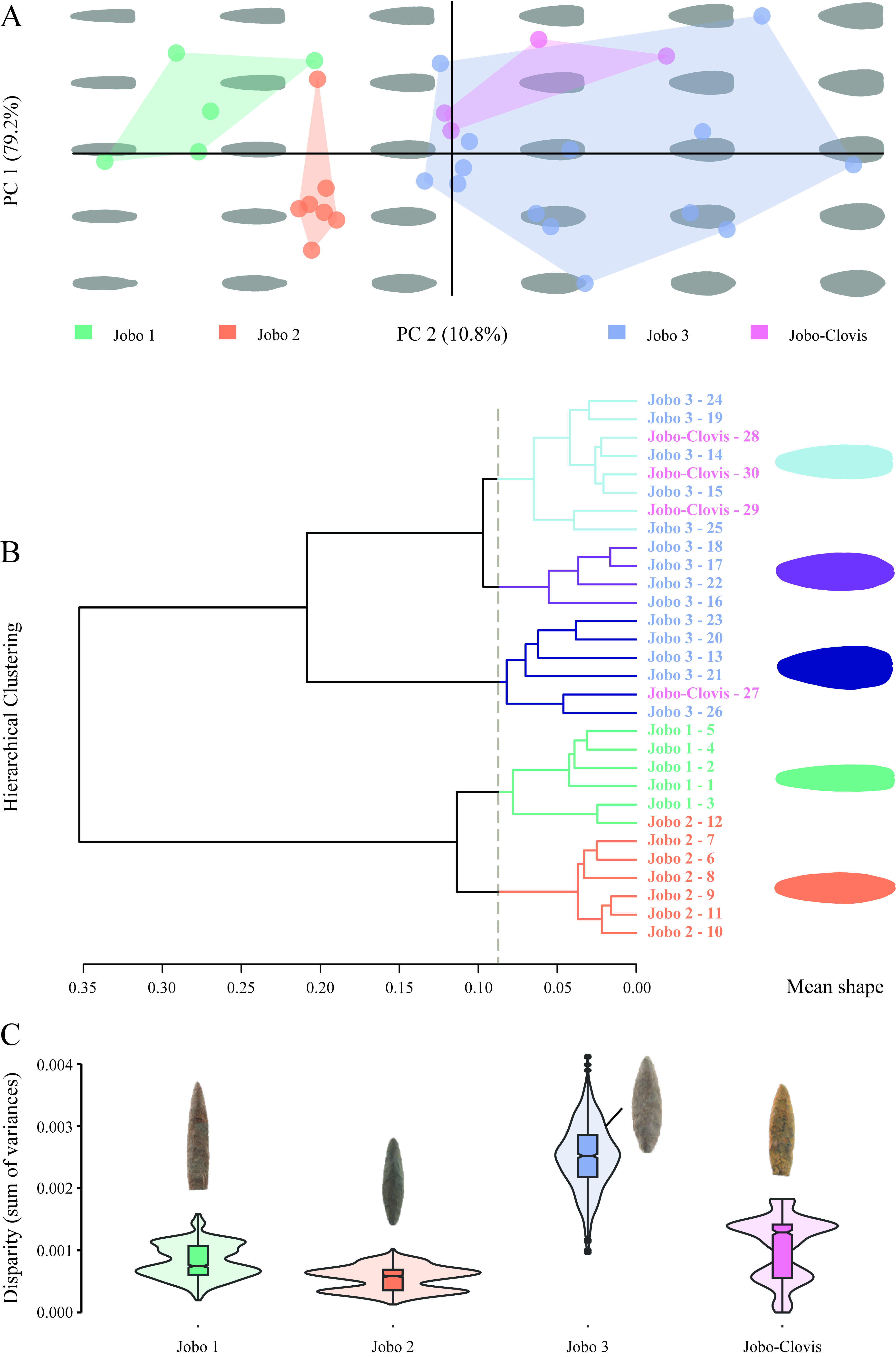

Supplement: Supplementary file 4 — High Resolution Image (TIF 2.85 MB) [file 12520_2025_2296_MOESM3_ESM.tif]
